# A Paper on Epidemics

**Published:** 1867-01

**Authors:** H. Nance

**Affiliations:** Kewanee, Ill.


ARTICLE II.

### A PAPER ON EPIDEMICS.

By H. NANCE, M.D., Kewanee, Ill.

Read to the Military Tract Medical Society.

Having been selected by this Association to report, at the semi-annual meeting, at Galesburgh, on Epidemics and Endemics, I owe an apology for not making this paper more interesting. I cannot make it interesting, for the good reason, that we really have not had a severe epidemic, of any disease, for two or three years. In taking a retrospect of the health of our community for the last two years, I would pronounce it unusually good. Our community, I think, have suffered much less than many others situated quite contiguous to us; this may be owing to the particular topography of our village and its environs. Kewanee is situated on the Chicago; Burlington, & Quincy Railroad, immediately on the dividing ridge between the Illinois and Mississippi Rivers. The south part of the village discharges its sewers into the tributaries of the Illinois River, and the north part enter the tributaries of the Mississippi River, thus leaving us almost entirely free from any stagnant pools of water. Physicians advocating a miasmatic origin of the usual autumnal fevers, would promptly come to the conclusion that we would suffer but little from this class of diseases, and this conclusion would be nearly correct.

Our village is underlaid with an immense field of bituminous coal, being from eight to ten feet below the surface to eighty or ninety feet, according to the grade of the land. How far this mineral or vegetable production may affect the health of our community, I shall not pretend to say; I will only remark that the health of our mining population is equally as good, if not better, than that of the rural population, who are engaged in cultivating the soil. I think they are remarkably exempt from koino-miasmatic diseases. It is very unusual to see any of them suffering with intermittent diseases; and I can say the same of typhoid fever. I have resided here more than six years, and during that time I do not remember of having treated a case of typhoid fever in the house of any miner. In the fall of 1864, I treated about a dozen cases of genuine typhus fever in the mining region, but it was introduced by a patient brought from off a ship sick with the disease, and from this case the others originated.

Notwithstanding we are on the dividing ridge between the two great rivers, and that our county is nearly all underlaid with coal, which may have a modifying influence upon the various epidemics of our county, yet we occasionally have epidemics. We have had cerebro-spinal meningitis, typhoid fever, measles, pertussis, dysentery, and, probably, some other diseases prevailing as epidemics since I have resided in Henry County; and I should not fail to mention the general prevalence of intermittents, remittents, infantile diarrhœas, and general derangements of the alimentary canal of children, usually under two years of age. During the winter, when the weather is mild and the atmosphere is overloaded with a redundancy of moisture, pneumonia and bronchial affections always prevail. Phthisis pulmonalis is becoming a much more general disease than it was at the first settling of the country, and I am sorry to add, with all our improvements in medicines, including the vaunted remedies, *oleum jecoris anselli* and the *phosphites*, that tuberculosis advances as rapidly as it did before the introduction of these *lauded* "*specifics.*" I mention these remedies, not to entirely condemn them, but to put the young and inexperienced practitioner on his guard, not to place too much confidence in them when prescribing for his phthisical patient.

There is, doubtless, a tendency on the part of some of our leading physicians to eulogize the virtues of some of our medicines too much. This excess of praise of these remedies is confined principally to professors in our medical colleges, in order to create a reputation, not only for themselves but for their institutions. I am frequently disgusted in reading in our medical journals of some remedy being recommended so highly for some epidemic which is prevailing at the time. Who of you now believe that the phosphites will eradicate tuberculosis, renovate the general system, and make a healthy, athletic man of a poor anæmic person? Who of you now believe that cod-liver oil is a specific in all pulmonary diseases? I tell you, gentlemen, that we should not be so easily deceived by pretenders in medicine who are trying to build themselves up, nor by professors in our medical colleges who are using every effort, not only to enhance their own reputations, but the reputation of the institution they represent. Look guardedly to the interests of your patient, and let us not vie with those who would rise to fame in their profession, regardless of the good they may do in curing their patients.

We would not regard consumption as either an endemic or epidemic disease, but as it frequently becomes a sequel of bronchitis, pneumonia, pleuritis, etc., especially in persons possessing a hereditary taint, I mention it in this connection. Since the introduction of auscultation and percussion by LAENNEC and LOUIS, we have had but little difficulty in diagnosing pulmonary diseases. At the early stage of the formation of tubercle, some difficulty may be experienced, but when we look to the general symptoms, including quick pulse, general wasting of the fatty tissues, cough, especially in removing the clothing on going to bed, occasional night sweats, and repeated attacks of hæmoptysis, we may most certainly come to the conclusion that our diagnosis will be correct if we call the disease consumption. But when we have properly applied the stethoscope, or used immediate auscultation, a doubt no longer remains.

I would dismiss the subject of phthisis, by hastily mentioning the treatment in general. Out-door exercise, when the weather is pleasant; good, rich diet, including meat of various kinds, also eggs, butter, and milk; I would give my patient but little medicine if he was comfortable; treat the urgent symptoms as they arise; if the cough troubles him much, give him a mild expectorant combined with an opiate—the latter will do more good than all the expectorants treated of in the U.S. Dispensatory. My usual form for an expectorant in phthisis is, morphia sul. grs.vj., aqua ʒij., shake and add syrup ipecac. syrup tolu āā ℥ij., tinct. sanguinaria ℥j., mix, and take one teaspoonful every 20 or 30 minutes, until the cough is relieved. When hectic symptoms make their appearance, I find much advantage from quinine with aromat. sul. acid, given in the stage of apyrexia. The hæmoptysis, I would treat with acetate plumbi et sul. morphia, nit. potash, minute doses of tart. anti. et pot., or, sometimes, a teaspoonful of common salt, swallowed suddenly, will promptly arrest it. The patient should be kept quiet, with his head and shoulders raised up in bed, jugs of hot water to his feet, and sinapisms to the thoracic region; cold drinks should be used, and the patient cautioned not to exercise much, not to cough, only when involuntarily compelled to, and to avoid the habit which many phthisical patients have of *hawking.* My general treatment, if any is thought advisable, would, of course, be stimulating and tonic. I would permit the use of wine, good stock ale, and would not discourage the use of good cognac brandy and milk, made in the form of egg-nog or milk-punch. I have sometimes given, with apparent advantage, Nich's prep. cin. and ferri. But I would remark that I have but little confidence in any general treatment. Good warm clothing; flannels next to the skin, and the general hygienic rules which all physicians are supposed to understand, is the safest course, in my opinion, we can pursue in this dreadful disease. My own observation has taught me that patients treated on this plan have lived as long and suffered as little as those who have been the victims of Rushton & Clark's spurious cod liver oil, or Prof. Churchill's phosphites.

In leaving the subject of phthisis, I touch on other pulmonary diseases, and the first I mention is hooping-cough. This disease has prevailed somewhat as an epidemic for the last six or eight months; there have been no peculiarities attending it; most of the cases have been light and uncomplicated; but few cases have required the protracted visits of the medical attendant. My treatment in uncomplicated cases has been an expectorant composed of com. s. scillæ, with syrup tolu, and tinct. opii camph. It has been rarely necessary to administer any laxative or cathartic. I am down on all specifics for this disease. The idea advanced by some of our medical men, that cochineal, belladonna, nitric acid, and several other vaunted remedies, will cut short the disease we are considering, is all *bosh*, as the politicians say. Treat the disease as a pulmonary, self-limited one, and our rational is correct.

Complicated cases of hooping-cough are always serious, especially when connected with diseases of the brain. Arachnitis, with effusion, is apt to follow; then come spasms, general convulsions, and death. Our treatment should be shaped to meet these symptoms as they arise, and I would refer you all to diseases of the brain, for treatment under their proper heads. During the late epidemic I had the misfortune to have two such cases come under my care, both of which died. All the symptoms of arachnitis presented themselves before death, including severe convulsions, hæmaplegia, blindness, etc., etc.

Catarrhal fever, or acute bronchitis, prevails in this section of country every winter and spring. It is confined, principally, to children under eight or ten years of age. The prominent symptoms are dry, hacking cough, quick pulse, flushed face, dry and hot skin, white or yellow fur on the tongue, bowels usually constipated, slight mucus râle. If the disease is neglected it results in pneumonia; then we have added to the physical symptoms dullness on percussion, with complete bronchophony, or in milder cases crepital râle. Occasionally I notice rusty colored sputa, though this latter is very rare. My treatment is simple and successful. I very rarely loose a patient with it, and when this does occur it is when complicated with pneumonia, as the bowels are constipated, and the liver in an unhealthy state, which is evinced by the light or green colored operations. I give to a child, two or three years old, a laxative composed of grs. iv. hyd. clo. mite., rhei pulv. grs. xv., mag. calc. grs. v., mix and divide in chart No. 5, one every four hours, until the bowels move. After administering one or two of these powders I prescribe an expectorant composed of com. syrup scill. ℥iij., tinct. opi. camph. ℥j., mix and give from one-quarter to one-third teaspoonful every hour and a half. If the child has a high grade of fever, which is generally the case, I give one or two drops of the tinct. veratrum in each alternate dose. This treatment, with very little variation, I have found very successful in these catarrhal or pulmonic diseases, usually styled catarrhal fever, pneumonia, or acute bronchitis of children. Adults are very rarely thus affected, if they have catarrhal fever. It is usually severely complicated with pneumonia or acute bronchitis, and sometimes congestion of the lungs, making it a case of serious import, which should be met according to the type of the disease. The idea of treating all cases of pneumonia with quinine, or all cases with tart. anti. et pot., or with tint. ver. viride and venesection, epispastics, etc., is nothing but empiricism; and he who pursues such a course must expect to hear the church-bell tolling over his misfortunes, and he need not be surprised, in a short time, to find himself preparing to emigrate.

Congestion of the lungs rarely appears as an epidemic. Let it appear either sporadically or epidemically, it is always a serious disease, and much depends upon the good judgment of the intelligent practitioner to combat it successfully. At one time, it might require the free use of the lancet, and upon this, nearly alone, should we depend; at another time, as we might expect, the free use of quinine, or the more diffusible stimulants, carb. ammo., or wine, or even good brandy; sinapisms, or epispastics should be freely applied over the lungs. When the lips become purple, the mind inactive, coma and somnolency making their appearance, the breathing becoming stertorous, let the pulse be either full and slow, or quick and feeble, you may soon expect dissolution. There is one symptom, indicating nearly certain death, not confined entirely to pulmonary diseases, and that is the rising and falling of the *pomum Adami*. When you observe this symptom, let other symptoms be as they may, you can quite safely pronounce your patient in, or not far from, *articulo mortis*.

Twenty-one years ago, when I first entered the profession, pleuritis was quite a frequent disease, but it has grown less and less frequent, until it is almost unknown. The lancet and opium were our sheet-anchors. The disease, in my practice, hardly known any more. Can any of you assign a reason why?

In the winter of 1861–2, diphtheria made its appearance, not only in Henry County, but it became a general epidemic throughout a great portion of the United States, in fact, it was not confined alone to our hemisphere, but much of it prevailed in England, France, and many other portions of Europe. It is a disease *sui generis*, that is, it has many peculiarities belonging to itself, unlike any other disease. It has been said by some of our profession, that it is identical with scarlatina. I can see but little resemblance, letting alone the *identity*. The only thing in the disease that in the least assimilates scarlatina, is the inflammation of the tonsils and fauces, and this, to an acute observer, is easily diagnosed. Diphtheria is a sub-inflammatory asthenic disease, in which the nervous and circulatory systems are seriously involved. The small and rapid action of the pulse would indicate some serious lesion to the circulation, some general breaking down of the whole system. The general symptoms are indicative of some blood-poison, and such it seems to be. As is the case with other epidemics, so with this! We know not the cause. Why the *poison* should spend itself locally upon the uvula, tonsils, palate, and, sometimes, the glottis, epiglottis, and trachea, I leave to wiser men than I am to reason upon. That the nervous system participates largely in this disease, is known by the great debility and irritability, also by the condition the nervous system is left in, in the sequel; for it is not uncommon to find partial paralysis of the organs of deglutition, and we occasionally find partial or complete amaurosis. I have also, in a few instances in my practice, observed partial paraplegia. These sequelæ, under a continued use of quinine, the ferruginous preparations, or the more powerful tonic and tetanic *strychnia*, have usually yielded, though I have found it necessary to continue them for weeks, and sometimes several months.

As diphtheria is conceded by all to be an asthenic disease, and one of general debility, we would readily come to the conclusion that a tonic and stimulating course would be the *sine qua non* in its treatment, and such is my belief. When first visiting patients with diphtheria, I immediately place them upon large doses of quinine every two hours—if the patient is a child six or seven years old, I would give it from $1\frac{1}{2}$ to 2 grs. at each dose, and alternate with half a spoonful of the saturated solution of the chlorate of potash, requesting the patient to gargle the latter before swallowing each dose. If there is great debility, as evinced by rapid pulse, general pallor, sordes, etc., I would use brandy in conjunction with the quinine. In 24 hours' time, I have seen very marked effects from this treatment, and I attribute the improvement principally to the quinine. I believe it is worth everything else in the treatment of diphtheria. Of course I would not ignore the use of probangs and gargles, but would use the first very rarely indeed, and the latter prepared of some mild astringent or antiseptic. Tannic acid and capsicum, in the proportions of one drachm of each to one teacupful of water, makes a very good gargle; and if the patients cannot be taught to gargle, let them swallow it. In this way it will prove equally as effectual as if only used as a gargle, and not prove detrimental to the system.

The treatment recommended when diphtheria first made its appearance, of swabbing out the throat once, twice, or three times a-day with solution of argent. nitratum; muriated tinct. ferri; tinct. iodine; permanganate of potash, etc., etc., was, in my opinion a very bad one, and in many instances, I believe, resulted seriously. For if, by these means, we removed the pseudo-membranous formation, it only left a raw, granulating surface, ready, if the system was in the condition, to very soon produce another membrane probably worse than the one removed. Let me say to you, let the membrane alone until it becomes loose and nearly detached, and then, with a pair of forceps, we can easily remove it from the throat. The main point in the treatment of diphtheria is, to support the general system while the disease is preying upon it, and if we do our duty here, in most cases in a few days, we will have the happiness of visiting our patient in a convalescent state. Local external applications I hold to be of but little value, a flannel cloth moistened with ammonia or camphor liniment, or a piece of fat pork sprinkled with pepper and salt, are probably as good as anything.

In the winter of 1863–4, cerebro-spinal meningitis made its appearance in our county. The symptoms are so well known to most, if not all of you, that it is not necessary for me to rehearse them. I had six cases of the disease, well-marked, and out of this number five of them died. Their ages varied from fifteen months old up to ten years. The one that recovered was a young man, aged about twenty-one. He had the most aggravated symptoms, including opisthotonos, trismus, coma, entire loss of sensibility, including loss of speech, etc., etc., and these symptoms remained for five or six days, and yet under treatment he recovered.

I was called to three other patients during the time the epidemic was prevailing, with severe pain in the head and cervical region, face flushed, head drawn back, full and rapid pulse, delirium, etc. I immediately bled each one of them, and gave tinct. ver. veridi in full doses; at the same time ordered an active cathartic. Under this treatment they all recovered. The six cases first spoken of were not treated in this active antiphlogistic way, for the reason that their symptoms would not admit of such activity. They were treated with laxatives—quinine, belladonna, mild opiates, carb. amo., etc., etc., with stimulating liniments, epispastics, etc., to the cervico-spinal region. Notwithstanding such authority as Dr. DAVIS has highly recommended—belladonna in the treatment of cerebro-spinal meningitis—I cannot see the rationality of it, and should feel very much indisposed again to try it. You may urge me for my treatment in this disease. I can frankly reply that I have none. I should treat my cases according to the symptoms. If active symptoms prevail, indicating congestion of the brain, with full pulse, flushed face, etc., I should certainly use the lancet and ver. viride; but if the type is asthenic, a tonic and stimulating treatment would look most plausible. I am firmly of the opinion, knowing the pathology of cerebro-spinal meningitis, that it will never be treated with much success. How can we successfully combat an inflammatory disease of the brain and spinal marrow, and their membranes, especially when it is an epidemic, and supposed to originate from some unknown ærial cause? Epidemics of all kinds are usually more virulent than when the same disease occurs sporadically, and this remark applies especially to the one under consideration.

Typhoid fever, which has prevailed in Illinois for a great number of years, and has been so very fatal in many localities, has nearly disappeared from this section of country. How long this immunity may continue none of us can tell. I have seen but five well-marked cases of typhoid fever in the last two years. Two of them were patients of my friend, Dr. Scott. They came under my care for ten or twelve days, during his short absence in Minnesota. They were genuine cases, as marked by petechia, sudamina, tympanitus, hebetude, loss of memory, quick pulse, stupor, hæmorrhage, etc., etc.; in the sequal, abscess occurred in the perinial region. The other cases I saw in consultation with Dr. Smead, of Lafayette, in Stark County. One of them had hæmorrhage from the bowels, and immediately after convalesced. My treatment in typhoid fever is quite simple. If the case is mild, I simply give spts. mindereri and nitre; if the pulse is quick and full I would add two or three drops of ver. viride every two or three hours. When diarrhœa makes its appearance, I would use an emulsion of spts. turpentine, with tinct. opii; would continue this treatment through the disease if the diarrhœa remains. The patient should be well supported on milk-broth, wine, etc.; should the vital forces seem to be giving away, quinine, brandy, etc., are indicated. Typhoid fever is a self-limited disease, and the idea of breaking it up in a few days is untrue. Many empyrics, when this disease is epidemic, call nearly everything *typhoid*, and hence establish a reputation for treating this disease. I would particularly urge upon the young physician not to give much cathartic medicine. If a laxative is imperatively demanded, (which is rarely the case,) give about half a teaspoonful of oleun ricini, with from ten to twenty drops of oleum terebinth.; this quantity will rarely fail to operate sufficiently thorough. Typhoid fever being an enteric disease, or dothinenteritus, we can easily conceive that a cathartic or laxative would move the bowels very readily, and such is the case. I never gives laxatives so long as the patient feels easy and comfortable, if the bowels should remain unopened for several days.

A species of stomatitis has prevailed as an epidemic since early in the spring, confined to no particular age. It produces ulceration about the roots of the teeth, on the palate, tongue, and, in fact, all over the mucous membrane of the mouth. In some cases the system seems to sympathize, producing a loss of tone, strength, and approaching anæmia. In such cases I have used quinine and the preparations of iron. Locally I have found more good derived from penciling the gums and ulcers on the cheeks with muriated tincture of iron, than from anything else, though I have used, with much benefit, sul. cupri and nitrate of silver. Some cases I treated with chlorate of potash, as a wash, and gave it at the same time as a *blood purifier.* It has not only acted as an epidemic, but has seemed to me to be contagious, spreading from one member of the family to another, by using the same drinking utensil, or from the parents kissing their children. Many cases have been stubborn, but all have yielded to the above treatment.

It remains for me to treat of two more classes of diseases which prevail in our county occasionally as epidemics, which I shall treat of very succinctly (as my essay has already grown too long). I allude to the *exanthemata* and diseases of the *stomach* and *bowels.*

In the winter of 1862–3, rubeola prevailed very generally, as an epidemic, not only in Henry County, but, I believe, very generally throughout the State. I think I must have treated more than a hundred patients, and, amongst that number, several in my own family. The type was remarkably mild, and, consequently, my treatment, to correspond, was equally simple. Where no complications existed, I simply prescribed cool drinks, *ad libitum*, equal temperature of the room, and the patients to remain indoors for a week or ten days after the exanthema disappeared. I rarely gave even a dose of sul. magnesia. When the catarrhal symptoms were troublesome, I gave a simple expectorant, composed of com. syrup scillæ and tinct. opii camp. As a general thing, I discard the idea of prescribing laxatives in rubeola, for the tendency, in the sequel, is diarrhœa in nearly all cases. Under this kind of treatment, I had the satisfaction of consoling myself that all my patients recovered, but one, and he was a boy of 10 or 12 years old, who had been subject to epilepsy from early infancy. Congestion of the brain and, finally, arachnitis made its appearance, and death soon closed the scene.

We have occasional cases of variola every year, but our people are so well protected by vaccination that we need have no fears of its appearing as an epidemic. My treatment is very similar to what it is in rubeola, when uncomplicated, and, consequently, need not be rehearsed. I would especially enjoin upon all the profession the propriety of urging upon the people the benefit of vaccination and re-vaccination, until the system seems to be insusceptible to its effects. This course being rigidly pursued, it would seem that small-pox, in a few years, would be unknown. Would it not be well to urge upon our representatives the propriety of enacting a law compelling the vaccination of all persons?

Scarlatina has not prevailed as an epidemic in this section of country since the years 1857–8–9. It then appeared in a malignant form, and many died. I see sporadic cases of it every year, and it is at the present time prevailing to some extent, though in a *very* mild form. My treatment is remarkably simple, and, since I have been using it, very successful. I would urge upon the members of this Society to give it a fair trial. When called, I order a solution of carb. of ammo. in doses of from 3 grs. to 5 or 7, according to the age of the patient, every hour and a-half or two hours. This treatment I give, regardless of the rapidity of the pulse, or the redness of the surface, and I must say, that my success has been unequalled since I commenced this mode of treatment. I use gargles of capsicum and chloride of soda, with vinegar and water, or simply a saturated solution of chlorate of potash, discarding the use of the swab or probang.

Diarrhœas, with adults and children, make their annual summer and autumnal visits, but their treatment varies so much that time will not permit me to treat of them.

Dysentery prevailed as an epidemic in the summer and fall of 1864. In adults, I usually give at the commencement, a good-sized dose of sul. magnesia; if this fails to check the disease, I order a powder composed of sul. morphia, acetate plumbi, and minute doses of calomel given every three or four hours, alternating every day or two with a portion of oleum ricini or sul. magnesia. This treatment will rarely fail to cut the disease short. The treatment in children and infants is not so successful, and I would respectfully refer you to the books. I have seen infantile dysentery as fatal as epidemic cholera, according to the number of cases attacked. I know of no treatment that has been very successful in this fatal form of dysentery, and would respectfully urge this Society to give their views on this part of my report.

The dreaded epidemic, cholera, has not made its appearance in our village this season, and I have heard of but one case in the county, and that occurred at Galva. The gentleman came through Chicago during the night, arrived in Galva in the morning, and died the same night, or early the next morning.

Not having treated any cases of cholera during the present epidemic, I am poorly prepared to give my views; but, notwithstanding this, probably I am as well prepared as many of you, and, possibly, as well as many who have been treating a great number of cases. Who of you would apply *ice bags* to your patients in collapse with this disease? Who of you would give castor-oil to your patients when the bowels were already profusely running off and the stomach rejecting everything that is swallowed? Who of you would expect to cure your patients when in a state of collapse by the administration of spirits of turpentime and strychnia? And yet such is the treatment recommended by some of the most learned men in our profession. In brief, I have no time to dwell upon this *King of Terrors*. I would treat my patients, if found in the cholerine or diarrhœa stage, with small portions of calomel, acetate of lead, and sul. morphine, given every two or three hours, until the diarrhœa was arrested; would order strict quietude and rest, in the recumbent position; very light diet, and small quantity of drinks of any kind—probably small quantities of iced-water, or even ice itself might be considered preferable. A treatment of this kind will usually succeed, but if we are so unfortunate as to lose this valuable time, or our patient sinks at once into vomiting, purging, cramps, and collapse, what is to be done? "Echo answers, what?" Now the critical time has come, and most of such cases die, regardless of the various treatments which have been proposed and used. Opium, or sul. morphia, so valuable in the first, or diarrhœa, stage, now is of much less value; if it is now given in large doses, or small ones frequently repeated, we produce comatose symptoms, loss of general vital force, a predisposition to arachnitis, and it also enhances the chances for a consecutive fever being set up, in case the patient rallies from collapse.

In this stage, I would give tinct. capsicum, tinct. camphor, ess. mentha, ss., and chloroform, combined with a very small quantity of tinct. opii; this preparation I would administer after every spell of vomiting, and if the vomiting ceased, I would give it as often as the cramps, diarrhœa, and pain seemed to indicate. I should be very careful not to produce the narcotic effects of this opiate. Stimulating injections might, with propriety, be given. Sinapisms and dry warmth should be used freely. I would give but little, if any water, preferring my patients should use ice in small pellicles to quench the raging thirst.

